# FMO-guided design of darunavir analogs as HIV-1 protease inhibitors

**DOI:** 10.1038/s41598-024-53940-1

**Published:** 2024-02-13

**Authors:** Hathaichanok Chuntakaruk, Kowit Hengphasatporn, Yasuteru Shigeta, Chanat Aonbangkhen, Vannajan Sanghiran Lee, Tanatorn Khotavivattana, Thanyada Rungrotmongkol, Supot Hannongbua

**Affiliations:** 1 Program in Bioinformatics and Computational Biology, Graduate School, Chulalongkorn University, Bangkok, 10330 Thailand; 2 Center of Excellence in Structural and Computational Biology, Department of Biochemistry, Faculty of Science, Chulalongkorn University, Bangkok, 10330 Thailand; 3 Center for Computational Sciences, University of Tsukuba, 1-1-1 Tennodai, Tsukuba, Ibaraki 305-8577 Japan; 4 Center of Excellence in Natural Products Chemistry, Department of Chemistry, Faculty of Science, Chulalongkorn University, Bangkok, 10330 Thailand; 5 Chemistry Department, Faculty of Science, University Malaya, Kuala Lumpur, 50603 Malaysia; 6 Center of Excellence in Computational Chemistry (CECC), Department of Chemistry, Faculty of Science, Chulalongkorn University, Bangkok, 10330 Thailand

**Keywords:** HIV-1 protease, Darunavir analogs, Structure-based drug design, Fragment molecular orbital (FMO), Combined analog generator tool, Computational biology and bioinformatics, Drug discovery, Structural biology

## Abstract

The prevalence of HIV-1 infection continues to pose a significant global public health issue, highlighting the need for antiretroviral drugs that target viral proteins to reduce viral replication. One such target is HIV-1 protease (PR), responsible for cleaving viral polyproteins, leading to the maturation of viral proteins. While darunavir (DRV) is a potent HIV-1 PR inhibitor, drug resistance can arise due to mutations in HIV-1 PR. To address this issue, we developed a novel approach using the fragment molecular orbital (FMO) method and structure-based drug design to create DRV analogs. Using combinatorial programming, we generated novel analogs freely accessible via an on-the-cloud mode implemented in Google Colab, Combined Analog generator Tool (CAT). The designed analogs underwent cascade screening through molecular docking with HIV-1 PR wild-type and major mutations at the active site. Molecular dynamics (MD) simulations confirmed the assess ligand binding and susceptibility of screened designed analogs. Our findings indicate that the three designed analogs guided by FMO, **19–0–14–3**, **19–8–10–0**, and **19–8–14–3**, are superior to DRV and have the potential to serve as efficient PR inhibitors. These findings demonstrate the effectiveness of our approach and its potential to be used in further studies for developing new antiretroviral drugs.

## Introduction

The human immunodeficiency virus type 1 (HIV-1) is a retrovirus that progressively destroys essential white blood cells of the immune system called CD4 + helper T cells. According to the World Health Organization (WHO), the number of people living with HIV has risen to nearly 38 million worldwide by 2022^1^. Even though most infected people have no symptoms, they need to receive HIV treatment and antiretroviral therapy (ART). The guideline for ART emphasizes interrupting different vital stages in the viral life cycle as an essential key for HIV drug development, including non-nucleoside reverse transcriptase inhibitors (NNRTIs), nucleoside reverse transcriptase inhibitors (NRTIs), integrase strand transfer inhibitors (INSTIs), and protease inhibitors (PIs)^2^. HIV-1 protease (HIV-1 PR) is a homodimeric aspartic PR consisting of ninety-nine amino acids per monomer with a catalytic D25, and two flexible β-hairpin flaps that cover the active site^1^ (Fig. S1 in Supporting Information). The enzyme cleaves viral polyproteins at specific sites, contributing to the mature structure and function of HIV-1 proteins^3,4^. Blocking the PR active site by PIs is crucial to prevent the mature virion assembly, viral replication, and increased viral load. Today, ten PIs have been approved by the Food and Drug Administration (FDA)^5–7^. One of the most potential PIs currently recommended as second or third-line antiretroviral therapy is darunavir (DRV)^1^. It was developed from amprenavir and fosamprenavir by modifying 3-methyl tetrahydrofuran, since several studies suggest the prevalence of HIV-1 drug resistance on PIs^8^. Moreover, the increased dissociative half-life of DRV results in prolonged release of the drug in the body, indicated by its binding affinity higher than 100-fold compared to other PIs^9^. Although DRV is highly effective in most patients, the current ART is still inadequate as HIV-1 can develop drug resistance through an accumulation of PR mutations, including V82A, I84V, and L90M^10–12^. Major PR mutations including D30N, V32I, M46L, G48V, I50V, I54M/V, L76V, and N88S were also found in the Weber et al.^13^ and, Stanford University^14^ study according to their databases on HIV treatment resistance. Furthermore, many studies have reported that several HIV patients show DRV drug resistance against PRs^15^.

Quantum mechanical (QM) calculations have made structure-based drug design more effective by accurately predicting molecular and electronic properties, including effects missing in molecular mechanics (MM), such as electronic polarization, charge transfer, halogen bonding, and covalent bond construction, which are superior to MM force fields. The fragment molecular orbital (FMO) method offers faster computational speed than conventional QM calculations. It is suitable for calculating macromolecular systems by partitioning them into several fragments and calculating them in parallel^16,17^. The pair interaction energy (PIE) and decomposed analysis of PIE (PIEDA) provide the contributed interaction energy and key chemical groups of known drugs that could be used to search for novel potent inhibitors or be a clue for drug design^18–21^.

**Figure 1:**
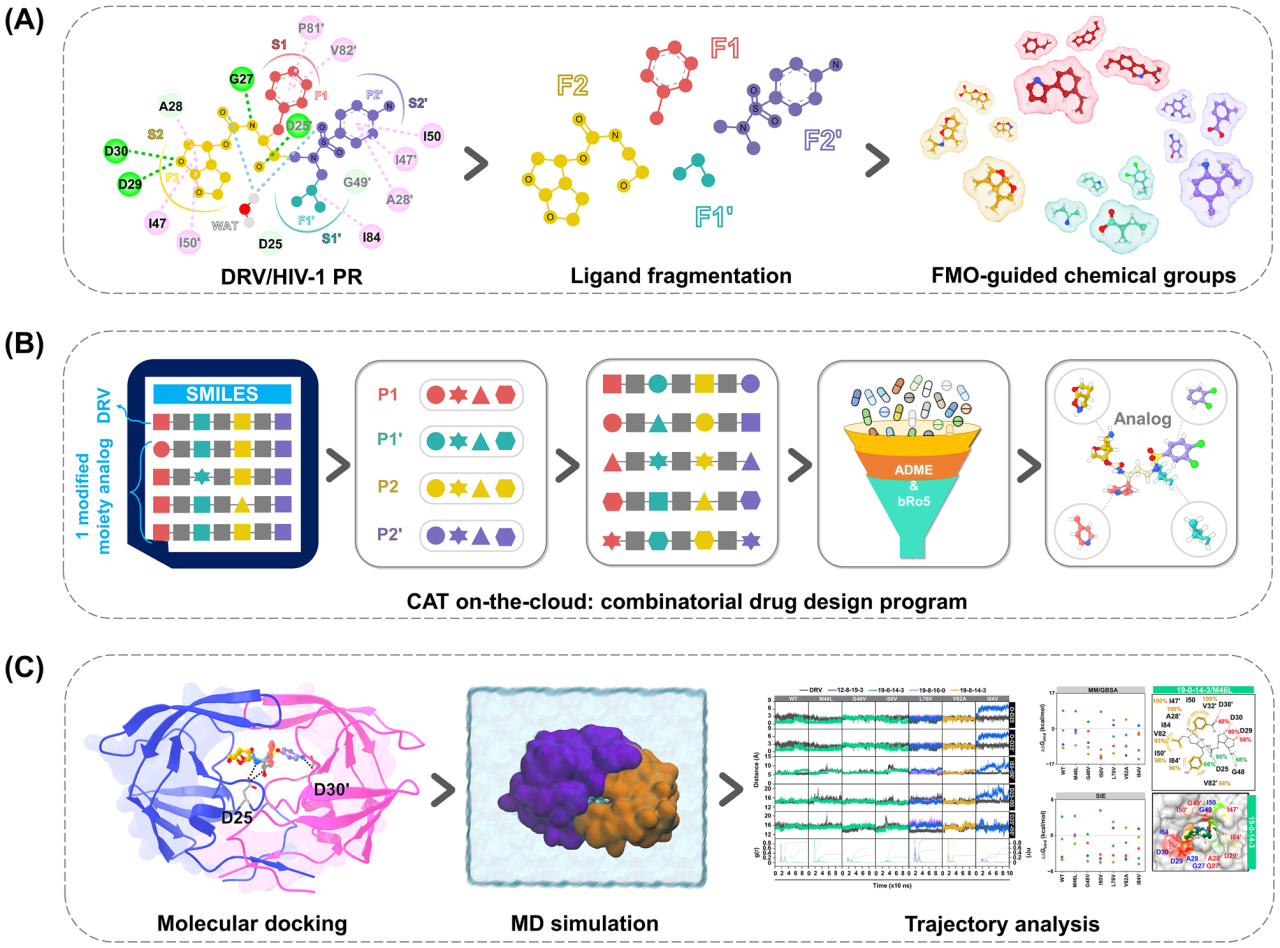
Workflow of the present study. (**A**) FMO-guided drug design in four fragments F1, F1′, F2, and F2′ of darunavir (DRV). (**B**) Combined Analog generator Tool (CAT)-generated analogs with favorable drug-like properties. (**C**) Molecular docking and molecular dynamics (MD) simulations of designed analogs.

The need for the commercially licensed software and standalone personal computers for substructure combination in structure-based drug design can pose significant challenges for researchers in terms of cost and accessibility^22–29^. This can limit the scope of research and hinder progress in developing effective antiretroviral drugs for diseases like HIV-1. Our study aims to develop a new approach that uses the FMO calculation to introduce the chemical substructure for cascade screening. The programming for combinatorial chemistry in drug design, Combined Analog generator Tool (CAT), was developed to build novel drug analogs with all possible combinations of substructures without requiring a license or expensive software. Initially, the FMO method was employed to investigate the ligand–protein interactions of the DRV/HIV-1 PR crystal structure complex. Based on the FMO results, substructure modification was carried out in four fragments (F1, F1′, F2, and F2′), as shown in Fig. 1A. These chemical substructures were considered as single-position modified DRV analogs and then screened using molecular docking. The potent chemical substructures were combined using CAT to create four-position combined DRV analogs. These were evaluated for ADME (absorption, distribution, metabolism, and excretion) prediction and beyond rule of five (bRo5) properties to identify potential drug-like analogs (Fig. 1B). After that, a set of four-position combined DRV analogs were docked into wild-type (WT) and major mutants (D30N, V32I, M46L, G48V, I50V, I54M, I54V, L76V, V82A, I84V, N88S, and L90M) HIV-1 PR, and the effective hits in most PRs were further analyzed using molecular dynamics (MD) simulations. The designed compounds’ structural dynamics, ligand–protein interactions, and predicted binding free energies were compared to those of DRV (Fig. 1C). The potential candidate analogs will be synthesized and tested for their biological activity in future studies.

## Materials and methods

### FMO calculation on DRV/HIV-1 PR complex

To analyze the binding energy of DRV/HIV-1 PR complex in terms of QM^30^, FMO calculation was performed using the DRV/HIV-1 PR crystal structure (PDB ID: 4LL3^31^), with a focus on amino acid residues within 7 Å of DRV. The General Atomic and Molecular Electronic Structure System (GAMESS) software^32^ was utilized for this purpose. In FMO calculation, the protein was typically fragmented into monomers by bond detachment atom (BDA) techniques^33^, which involved fragmenting the protein at the bond between Cα-C of the protein backbone. Based on this approach, the DRV molecular structure was divided into four fragments: F1, F1′, F2, and F2′ (Fig. 3A). PIEDA was computed using the second-order Møller–Plesset perturbation theory (MP2) with the resolution of identity (RI) approximation and polarizable continuum model (PCM) solvation effect (FMO-RIMP2/PCM) at the B3LYP/6-31G* basis set^34^. The crucial interacting residue for DRV binding was identified by computing the pair interaction of monomers (I and J) using the following equation.

$$PIE = {\Delta E}_{IJ}^{ES}{+ \Delta E}_{IJ}^{CT+mix}+{\Delta E}_{IJ}^{DI}+{\Delta E}_{IJ}^{EX}+{\Delta G}_{Sol}^{PCM}$$

The ligand binding interaction is denoted as the PIE term, and it comprises of the electrostatic interaction ${(\Delta E}_{IJ}^{ES})$, the charge transfer with higher-order mixed terms energies ${(\Delta E}_{IJ}^{CT+mix})$, dispersion $({\Delta E}_{IJ}^{DI})$, exchange-repulsion ${(\Delta E}_{IJ}^{EX})$, and the polarizable continuum model (PCM) solvation effect (${\Delta G}_{Sol}^{PCM})$^35–37^.

### Molecular docking study of designed analogs

In this study, cascade screening using molecular docking were performed with two sets of ligands: *(i)* single-position modified DRV analogs and *(ii)* four-position combined DRV analogs. In the first screening, a set of 20 chemical groups was designed for each substructure, namely P1, P1′, P2, and P2′, corresponding to the fragments (F1, F1′, F2, and F2′) computed through FMO calculations. A total of 80 single-position modified DRV analogs were created for the first molecular docking with WT HIV-1 PR. The crystal structure of WT HIV-1 PR in complex with DRV (PDB ID: 4LL3^31^) was prepared as the protein receptor for the molecular docking study. The DRV ligand was removed, and hydrogen atoms were added. The protonation state was assigned using PDB2PQR web server^38^, except for D25 and D25′, which were assigned according to the previous studies^39^. Molecular docking study was conducted using the GOLD program with genetic algorithm (GA)^40^. The docking site was defined as a 10 Å sphere centered at DRV, located in the active site of HIV-1 PR. To validate the system, redocking of DRV was performed and compared to the crystallized ligand, resulting in a root mean square deviation (RMSD) of 0.65 Å (as depicted in Fig. S3 in the Supporting Information).

After the first round of molecular docking, the single-position modified DRV analogs with higher binding affinity scores compared to DRV were chosen to construct the four-position combined DRV analogs (see detail in section "Combined analog generator tool" Combined analog generator tool of “Materials and methods”). These analogs were then subjected to the second-round molecular docking that focused on both WT and mutant systems. A total of 12 major HIV-1 PR mutations, including D30N (PDB ID: 7DOZ^41^), V32I (PDB ID: 2HS1^42^), M46L (PDB ID: 2HS2^42^), G48V (PDB ID: 3CYW^43^), I50V (PDB ID: 6DH6^10^), I54M (PDB ID: 3D1Z^43^), I54V (PDB ID: 3D20^43^), L76V (PDB ID: 3PWM^44^), V82A (PDB ID: 2IDW^45^), I84V (PDB ID: 2IEO^45^), N88S (PDB ID: 3LZU^46^), and L90M (PDB ID: 6OOS^46^), were prepared as the receptor according to the previously described method. The interactions between the ligand and protein were visualized using the UCSF Chimera package^47^ and Accelrys Discovery Studio 3.0 (Accelrys Inc.)^48^.

### Combined analog generator tool

**Figure 2:**
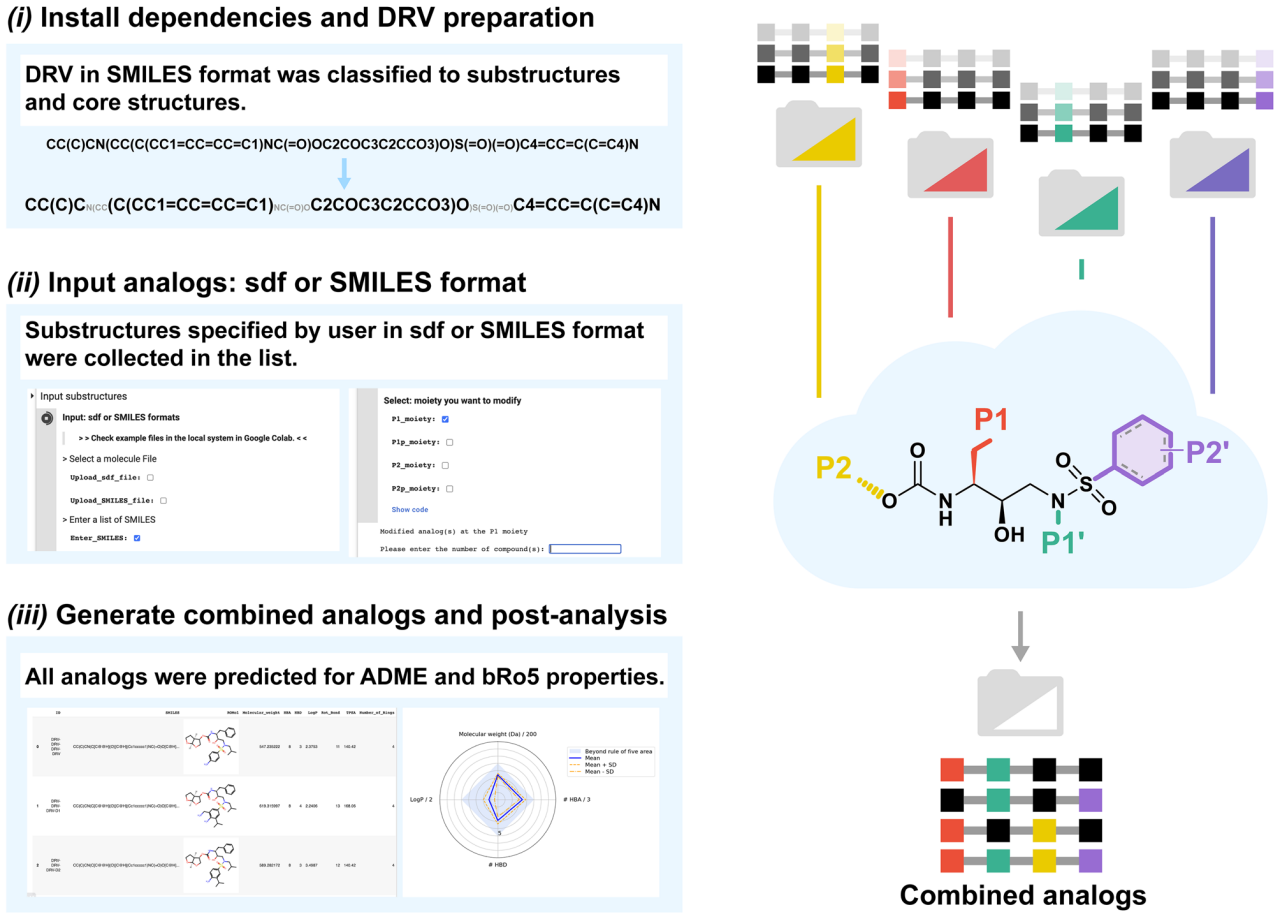
Workflow of Combined Analog generator Tool (CAT) (Fig. S2 in Supporting Information).

The four-position combined DRV analogs were generated using a Python-based combinatorial algorithm called the CAT^49^, implemented in Google Colab^50^, The process involved three steps: *(i)* installing dependencies and preparing DRV, *(ii)* preparing single-position modified DRV analogs, and *(iii)* generating combined analogs and performing post-analysis (Fig. 2). Initially, DRV and potent single-position modified DRV analogs were identified for core structures and substructures (P1, P1′, P2, and P2′) using Open Babel version 3.1.1.16^51^. The list of selected potent single-position modified DRV analogs for each position (P1, P1′, P2, and P2′) was uploaded to Google Colab. These analogs were then combined in all possible combinations based on the core structure of DRV and converted to 3D structures using the RDKit package^52^, employing the ETKDG method^53^. Ligand conformers were created, aligned, and minimized using the Universal Force Field (UFF)^54^. Additionally, the physicochemical features, including ADME and bRo5 properties of the analogs, were also predicted by the RDKit package^52^.

### Molecular dynamics (MD) simulations

To prepare the system for all-atom MD simulations, the AMBER ff19SB force field was applied to the protein, while the ligands for this step were optimized by the HF/6–31d level of theory using the Gaussian 09 program^55–57^ and applied for the General AMBER Force Field 2 (GAFF2)^58^, as the standard protocol^56,57,59^. To add any missing hydrogen atoms, the tLEaP module was employed, followed by energy minimization using steepest descent (SD) and conjugated gradient (CG) methods with 1,500 steps to eliminate any improper contacts. Counterions were then introduced to neutralize the system, and the TIP3P water model was used to immerse the system in an octahedral box that extended at least 10 Å from the protein surface^60^. Energy minimization was performed on the explicit water and neutralized ion molecules using SD and CG methods with the same iterations. During the MD simulations, nonbonded interactions were cutoff at 10 Å for short-range interactions, and the Particle Mesh Ewald (PME) summation approach was used for long-range electrostatic interactions^61,62^. All covalent bonds involving hydrogen atoms were constrained using the SHAKE algorithm, and temperature and pressure were controlled. The simulated models were heated from 10 to 310 K over 100 ps and held at this temperature for 100 ns under periodic boundary conditions with an isobaric-isothermal (NPT) ensemble, implemented in the AMBER 20 package program^58^. The last 20 ns-MD trajectories were used to analyze in terms of the structural dynamics of the complex using the CPPTRAJ module^63^. The binding affinity between the ligand and protein was then calculated using two methods: MM/GBSA^64,65^ and SIE^66^.

## Results and discussion

### FMO-guided design of drug analogs

**Figure 3:**
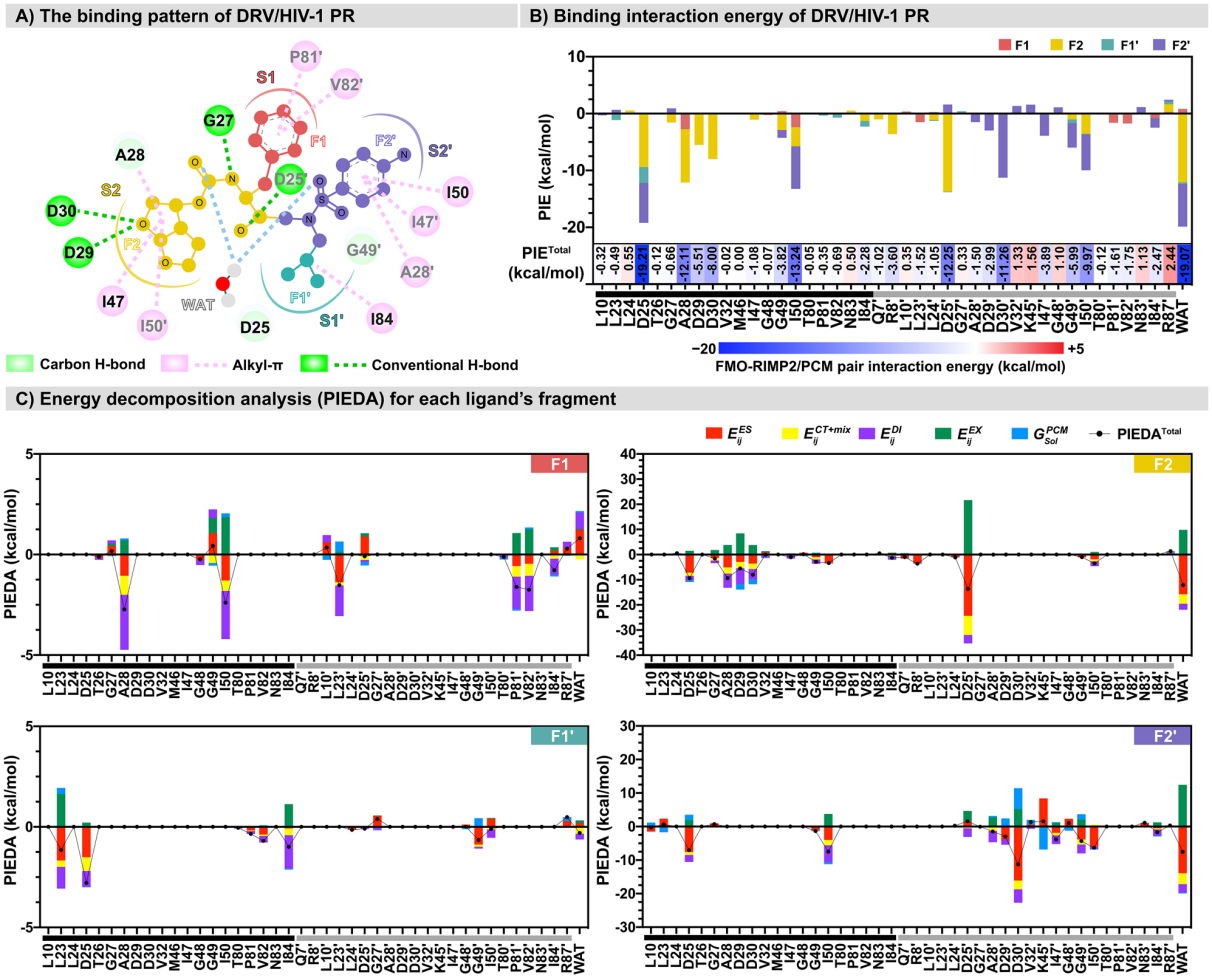
(**A**) The 2D intermolecular interactions of DRV binding to the active site of HIV-1 PR were obtained from FMO-RIMP2/PCM calculations. (**B**) The pair interaction energy (PIE) of the four fragments of DRV interacting with four subsites, S1, S1′, S2, and S2′, of HIV-1 PR was plotted as a stacked bar. (**C**) The decomposition free energy analysis (PIEDA) of each fragment, F1, F1′, F2, and F2′, was independently represented as a bar graph. Residues on chains A and B were shown in black and gray, respectively.

The structural-based drug design of DRV analogs was guided by an insight into DRV/HIV-1 PR interactions (Fig. 3A) resulting from the FMO-RIMP2/PCM calculation. The DRV molecular structure was divided into four fragments, F1, F1′, F2, and F2′, by covalent bond fragmentation at sp^3^ carbon atoms, and named according to their interacting PR subsites: S1, S1′, S2, and S2′. The drug/protein interactions were described in terms of PIE and PIEDA in Fig. 3 and the supporting Table S1. FMO-RIMP2/PCM detected 42 residues and the bridging water involved in the DRV/HIV-1 PR complex (Fig. 3B). The result of the PIE suggested that the primary interaction of DRV resulted mainly from the fragments F1, F2, and F2′, while the smaller fragment F1′ showed a lower contribution to the overall binding energy due to the size of fragment. Some residues provided stabilization of more than one fragment, such as catalytic D25 (− 19.21 kcal/mol for F1′, F2, and F2′) and I84 (− 2.28 kcal/mol for F1′ and F2), which are an important residues^39^. Previous studies have reported that the V82T/I84V mutation prevents the closed conformation of flaps and causes DRV resistance^10,12,67,68^ (Table S1 in Supporting Information).

**Figure 4:**
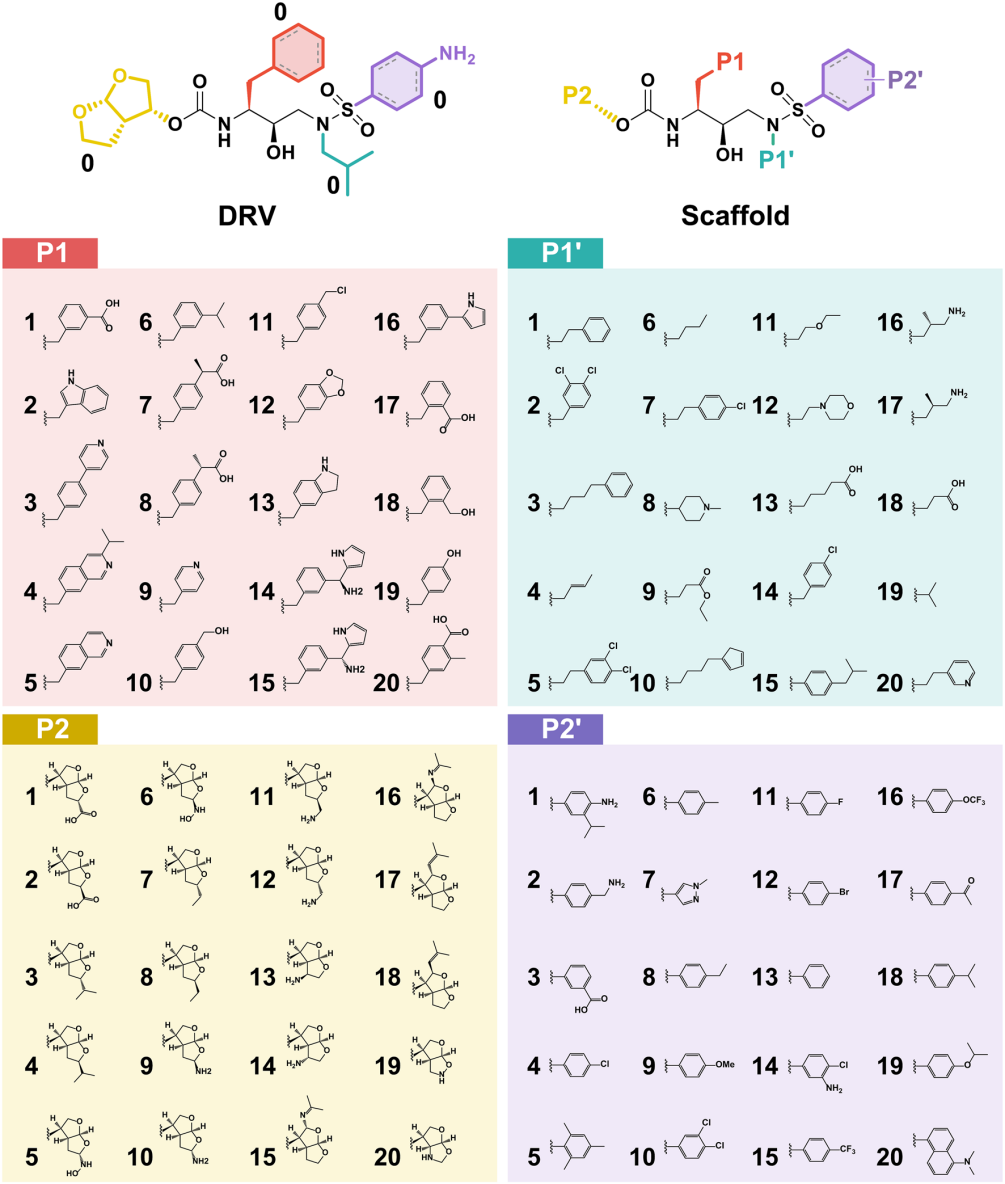
2D structures of DRV and the scaffold of DRV analogs with different substitutions on the P1, P1′, P2, and P2′ positions, where the chemical structures of modified functional groups (1–20) at each position are shown below.

The PIEDA for all four fragments was plotted separately to reveal the energy components of interaction (Fig. 3C) and used to guide for the chemical substructures in each moiety (Fig. 4). Hydrogen bond (H-bond) interaction and salt bridges refer to the electrostatic (${\Delta E}_{IJ}^{ES}$) and charge transfer with higher-order mixed terms energies (${\Delta E}_{IJ}^{CT+mix}$), while hydrophobic interaction can be suggested by dispersion (${\Delta E}_{IJ}^{DI}$). The steric repulsion between atoms was described by the exchange-repulsion energy (${\Delta E}_{IJ}^{EX}$)^21,69^. Our selection of substructures used at various substitution sites (P1, P2, P1′, P2′) was elucidated based on guidelines from previous studies. The aim in designing each substitution was not to replicate similar substructures from previous studies; instead, we endeavored to modify their substructures to achieve better interactions with HIV-1 PR. Considering that F1 interacts with the S1 subsite, a relatively good PIEDA showed that A28, I50, L23′, P81′, and V82′ mainly interacted with F1 through hydrophobic interaction (${\Delta E}_{IJ}^{DI}$). However, the high ${\Delta E}_{IJ}^{EX}$ found in catalytic I50 could affect the system. To increase the diverse interactions of DRV’s P1, Gordon’s research indicated that the DRV analog (GS-8374), which incorporates a phosphonate group with an aromatic core, enhances van der Waals (vdW) interactions with PR^70^. In our study, we modified substructures by adding hydroxyl (substructures 10, 18, and 19), carboxylate (substructures 1, 7, 8, 17 and 20), and amino groups (substructures 2, 13, 14, 15 and 16) to the aromatic core to achieve more robust hydrogen bonding with the hydrophobic residue. Additionally, the replacement of the P1-phenyl group with two fluorine atoms to create bis-fluoro-benzyl has been shown to introduce halogen bonds with PR and increase lipophilicity against PR mutant^71^. Therefore, the halogen group in the substructure was changed to chlorine (substructure 11) to introduce halogen bonds more effectively. The ${\Delta E}_{IJ}^{DI}$- associated hydrophobic forces were essential for the interaction of the F1′ sec-butyl moiety of DRV with L23, D25, and I84. Most of the substructures in the P1′ moiety were modified to larger hydrophobic groups (such as a hydrocarbon chain) in comparison to DRV. This modification targeted both WT PR and variants with main mutations^10,68,72^, aiming to enhance potency in cellular assays compared to DRV^10,70,72^. Hence, a larger hydrocarbon (substructures 4, 6, 9, 11, and 13), along with the incorporation of halogen (substructures 2, 5, and 14), carboxylate (substructures 13 and 18), and amino groups (substructures 16 and 17), was also considered in designing the substructures in this moiety to raise electrostatic attraction. Moreover, the substitution with aromatic rings, such as substructures 1, 5, 8, and 20 at P1′, had not been previously investigated. Intriguingly, in our study, both an aromatic ring and a hydrocarbon chain were designed for this moiety to increase hydrophobic interactions.

In addition to the H-bond formation with D25′ in the core structure of DRV's F2, the *bis*-tetrahydrofuranyl (THF) urethane was likely stabilized by A28, D29, D30, I47, and I50′. Designing additional groups, such as *tris*-THF urethane, methoxy, and *gem*-difluoro at P2 moiety to introduce H-bonds with the conserved main chain atoms of the PR (D29, D30, G27, and G48), which cannot be easily altered by mutations, was supported by previous findings^73,74^. Consequently, our designed substructures were modified by adding hydroxyl (substructures 5 and 6), carboxylate (substructures 1 and 2), and amino groups (substructures 9 to 14) to the *bis*-THF moiety to increase interactions with additional residues. The *bis*-THF urethane was retained for P2 substructures, while the hydroxyl, carboxylate, and amino groups were introduced to strengthen H-bonds with D25, D29, D30, G48, D25′, G27′, and G49′. The PIEDA of F2′ revealed that CH3-π and electrostatic interactions were important for the 4-methylaniline moiety. Antiviral DRV analogs (GRL-04410 and GRL-0519) have a methoxy substitution at P2′, as previously described by Weber and co-workers^73^. Modifications with methyl, halogen and isopropyl groups at this position improved the antiviral activity of the analogs^74,75^. In our study, to expand hydrogen bonding, hydrophobicity, and electrostatic attraction, we added halogen (such as meta and para-dichloro, para-fluoro in substructures 10, 11, and 12), isopropyl (substructure 18), isopropyl ether (substructure 19), and amino (substructures 1, 2, and 14) groups to the original DRV aromatic ring. The DRV analogs from an earlier study revealed that the modification at P2′ using a carboxylate group maintained polar contact and exhibited exceptional potency in an enzymatic assay^76^. Thus, the introduction of carboxylate and amino groups was designed in this moiety to enhance H-bond interactions. Adding a halogen atom or amino group to this fragment could increase the hydrophobic interaction with A28′ and I47′ and electrostatic attraction with D25, I50, D29′, D30′, I47′, G49′, and I50′ through ${\Delta E}_{IJ}^{ES}$ and ${\Delta E}_{IJ}^{CT+mix}$. The introduction of carboxylate and amino groups enhances the H-bond interaction with residues D29′, D30′, and G48′.

### Cascade screening

In this study, we introduce an approach utilizing the FMO method to guide the design of chemical substructures with the aim of developing analogs of DRV. The cascade screening strategy employed molecular docking to evaluate two sets of DRV analogs: *(i)* single-position modified analogs and *(ii)* four-position combined analogs. Specifically, we designed a comprehensive library of 80 single-position modified DRV analogs, with 20 analogs per position suggested by the FMO calculation. Subsequently, we conducted molecular docking simulations to predict the binding energies of these analogs with the WT HIV-1 PR protein, comparing them to the original DRV as a baseline during the initial screening phase. Notably, our results revealed significant improvements in binding energies for several chemical substructures compared to the original DRV compound. A molecular docking study revealed that positions P1, P1′, P2, and P2′ contained 5, 4, 8, and 3 potent substructures, respectively, which demonstrated favorable binding properties. As a result, these promising substructures were selected for further investigation. To explore their therapeutic potential, we expanded upon these potent chemical substructures by constructing an extensive library of four-position combined DRV analogs, comprising a total of 1,080 analogs (Fig. S5 in Supporting Information). Subsequently, we performed molecular docking experiments to assess the binding energies of these analogs against both the WT HIV-1 PR protein and 12 mutants.

To create the four-position combined DRV analogs, we developed a user-friendly and efficient combinatorial program using a Python script that was implemented in Google Colab, CAT. These substructures were then combined in all possible positions using the combinatorial programming algorithm (Fig. 2). The pharmacological properties were investigated for drug-likeness in terms of molecular weight (MW), the number of H-bond donors (HBD) and acceptors (HBA), polar surface area (PSA), and lipophilicity (LogP) using the RDKit package^77^ implemented in Google Colab. This is one of the features included in our proposed program in this study. The results suggested that all designed analogs and DRV were well within the bRo5 criteria, including *(i)* MW ≤ 1000 Da, *(ii)* HBD ≤ 6 and HBA ≤ 15, *(iii)* PSA ≤ 250 Å, and *(iv)* LogP ≤ 10^78^ (File2 in Supporting Information).

**Figure 5:**
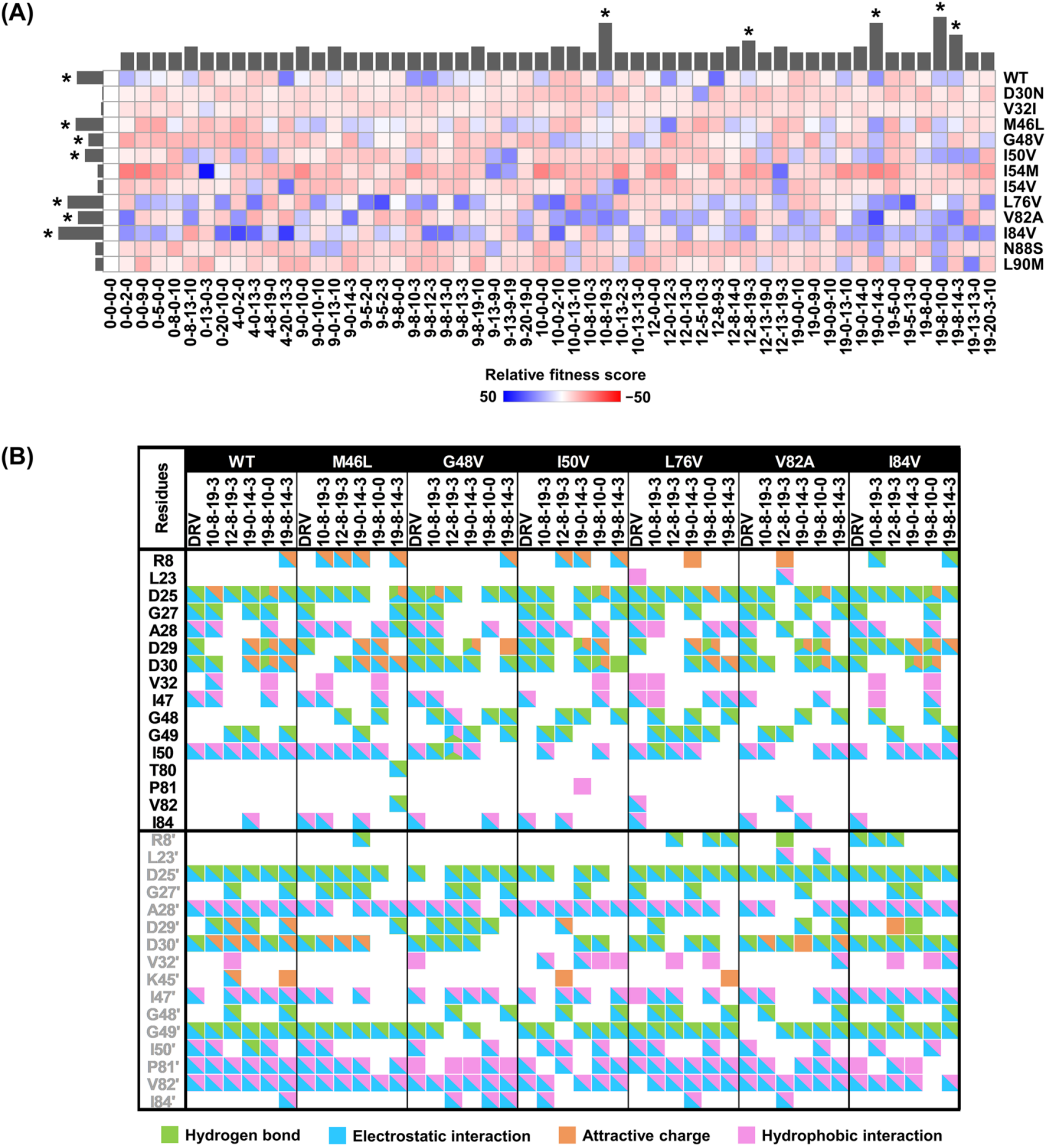
(**A**) the relative fitness scores of DRV (0–0–0–0) and selected analogs compared to the fitness score of DRV, as demonstrated by the grid map and collective data bar plot in the above and right panels. The potent analogs, ranked by fitness scores and marked with an asterisk (*), are also presented. (**B**) the interactions between the potent analogs and HIV-1 PR are depicted in the grid map using various colors to indicate different types of contact residues. For example, green, blue, orange, and pink colors represent H-bond, electrostatic interaction, attractive charge, and hydrophobic interaction, respectively.

To investigate the binding mechanism of DRV and generated analogs towards the binding site of WT HIV-1 PR and 12 major mutated PR strains (D30N, V32I, M46L, G48V, I50V, I54M, I54V, L76V, V82A, I84V, N88S, and L90M). These compounds were docked into the binding site of WT HIV-1 PR and 12 mutants using the GOLD program. All analogs in each system were then calculated to determine which analogs provided high fitness scores frequently in overall WT and mutants compared to DRV. Afterwards, the strains with high frequency found in a group with high fitness scores were selected for further investigation. The grid map in Fig. 5A shows five potent DRV analogs, including **10–8–19–3**, **12–8–19–3**, **19–0–14–3**, **19–8–10–0**, and **19–8–14–3**) with a higher fitness score (blue) than DRV in WT and most mutations. The fitness scores of these five analogs are shown in Table S2 in the Supporting Information.

By observing the common structural features of P1 and P1′ from the five potent analogs, it was found that the original DRV, namely 4-hydroxybenzyl for P1 (**19**–x–x–x) and 1-methylpiperidin-4-yl for P1′ (x–**8**–x–x), were the most effective. In addition, some other variations, such as introducing benzyl and methyl groups as 4-(hydroxymethyl)benzyl and benzo[d][1,3]dioxol-5-ylmethyl for P1 (**10**–x–x–x) and (**12**–x–x–x), respectively, or introducing a phenyl group for P1′ (x–**0**–x–x), could lead to favorable interactions with HIV-1 PR. Furthermore, for P2, all of the five analogs benefited from introducing the (4*R*)-amino group as (3*S*,3a*R*,4*R*,6a*S*)-4-aminohexahydrofuro[2,3-b]furan-3-yl (x–x–**14**–x) and hydroxyl hydrogen group as (3a*R*,4*S*,6a*S*)-hexahydrofuro[3,2-d]isoxazol-4-yl (x–x–**19**–x), suggesting a critical interaction in this position. Lastly, introducing a hydrophobic carboxyl group as 3-carboxyphenyl (x–x–x–**3**) seems to be the most common feature in the five analogs for P2′ modification.

Due to the preference for aromatic residues, strong hydrophobic interactions were observed at positions P1 and P1′ with I50, I84, and I84′ for P1 and L23, V82, and I50′ for P1′ (Fig. 5B). These positions correspond to the modeled peptide position in positions P1′ and P1 of the PR-peptide complex^79^ inserted into S1′ and S1 subsites. Previous studies have supported the design of cyclic, acyclic, and aromatic non-polar *N*-alkoxy moieties at the P1′ position^76,80^, similar to the P1 position suggested by FMO calculation in our study. Novelty, this result suggests that the preferable chemical moiety for S1′ and S1 subsites should exhibit hydrophobic properties, while the S2 and S2′ subsites (half P2 and P2′) can accommodate both polar and hydrophobic effects^81–85^.

To evaluate the binding interactions of the most promising DRV analogs, GOLD docking provided information on several interactions, including H-bonds, electrostatic interactions, and hydrophobic interactions. By comparing them to DRV, we found new interactions among our designed analogs in residues *(i)* G48 and I84 on both chains, *(ii)* R8, V32, G49, T80, P81 on chain A, and *(iii)* L23′, D29′, K45′, I84′ on chain B. The results verified that the FMO-guided design could be advantageous for introducing plausible chemical groups to enhance binding interactions^21^. However, the effective five analogs were selected for MD simulations to evaluate the physical movements of atoms and molecules of complexes.

### MD–based screening via binding free energy

**Figure 6:**
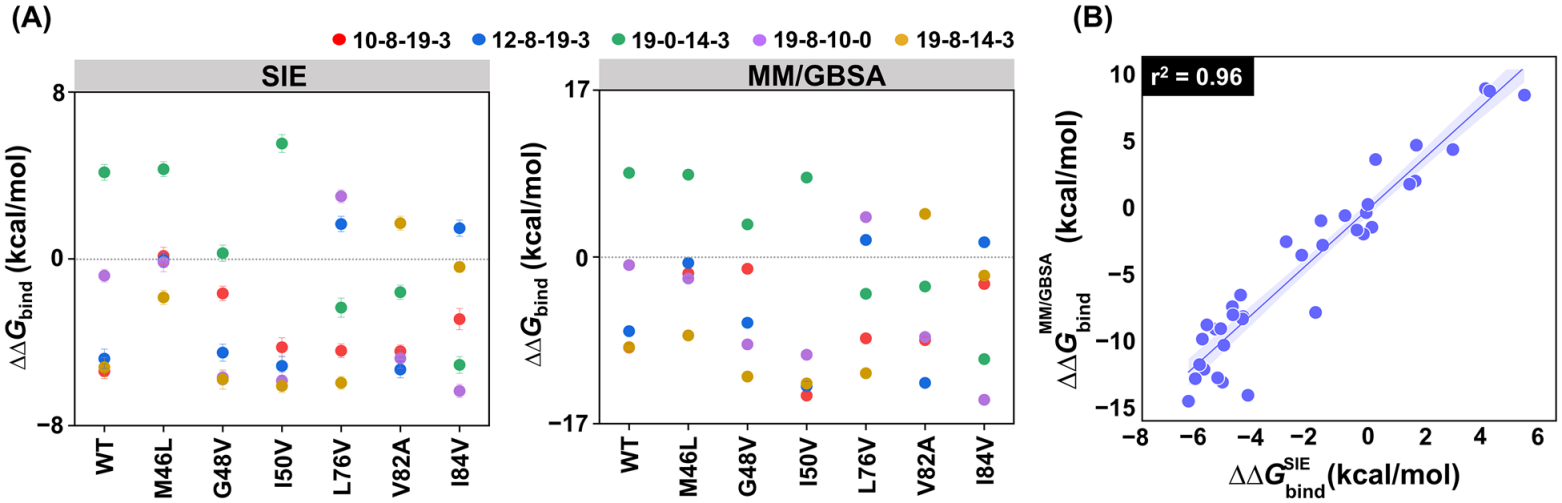
(**A**) The results of Δ*G*_*bind*_ (kcal/mol) based on the SIE and MM/GBSA methods are shown as the mean ± standard error of the mean (SEM) of simulations (**B**) Pearson correlation coefficient (r^2^) analyses between SIE and MM/GBSA are plotted. ${{\Delta\Delta G}}_{bind}^{DRV}$ illustrated by the dash line as a standard ΔΔ*G*_*bind*_.

To evaluate the efficacy of various analogs in both WT and mutant systems, the dynamic and solvation effects were taken into consideration. To accomplish this, a total of 42 complexes, consisting of six screened compounds and seven proteins, were subjected to MD simulations. The binding affinity of DRV and its analogs to HIV-1 PRs was assessed using the SIE and MM/GBSA methods on 100 snapshots taken from last 20 ns-MD trajectory. The last 20 ns of the simulation was selected for binding affinity calculation because every system reached an equilibrium state during this period, making these trajectories suitable for analysis. The binding energy (${\Delta G}_{bind}$) of screened analogs was compared to DRV by considering the change in binding free energy values (${{\Delta\Delta G}}_{bind}$ = ${{\Delta G}}_{bind}^{DRV}$ − ${{\Delta G}}_{bind}^{analog}$) in WT and mutated systems (Fig. 6A). If ${{\Delta\Delta G}}_{bind}$ has a positive value, it indicates that the analog binds to PRs better than the DRV system. Conversely, a negative value of ${{\Delta\Delta G}}_{bind}$ suggests that DRV binds to PRs better than the analog system. When ${{\Delta\Delta G}}_{bind}$ is zero, it indicates that the analog binds to PRs comparably with the DRV system. Furthermore, as part of the initial screening process to identify potent DRV analogs against both WT and mutated HIV-1 PR, 100-ns MD simulations were conducted in a single run. The reference DRV was also subjected to 100-ns MD simulations in triplicates, and the binding affinity was calculated to assess the consistency of the results, as illustrated in Figure S6 and Table S3 in the Supporting Information. The result suggested that four analogs (**12–8–19–3**, **19–0–14–3**, **19–8–10–0**, and **19–8–14–3**) in seven systems (WT, M46L, G48V, I50V, L76V, V82A, and I84V) could be the most promising compounds for PIs. Notably, the ${{\Delta\Delta G}}_{bind}$ values obtained using both methods exhibited a high correlation with r^2^ = 0.96 based on Pearson correlation^86^ (Fig. 6B).

According to ${{\Delta\Delta G}}_{bind}$, the **19–0–14–3** could be the common potent analog that effective against WT and the other three vital mutations: M46L, G48V, and I50V. While **12–8–19–3** could bind to the L76V and I84V systems. The analogues **19–8–10–0** exhibited higher binding affinity and inhibitory efficiency than DRV against only the L76V mutation, while **19–8–14–3** demonstrated good binding free energy to V82A mutations. The relationship between the five potent analogs was associated with combined critical substructures in two patterns: *(i)* sharing the chemical moiety at P1, P2, and P2′, such as **19–0–14–3** and **19–8–14–3**, and *(ii)* sharing the chemical moiety in P1′, P2, and P2′, such as **10–8–19–3** and **12–8–19–3**. Notably, chemical substructure 3 of P2′ moiety was shared in both analogs, thus indicating that this optimal chemical group may be the most important for developing DRV analogs. Consequently, distance analysis and radial distribution function (RDF) were performed to estimate the potency of designed substructures, including analogs.

### Ligand/protein stability evaluation

**Figure 7:**
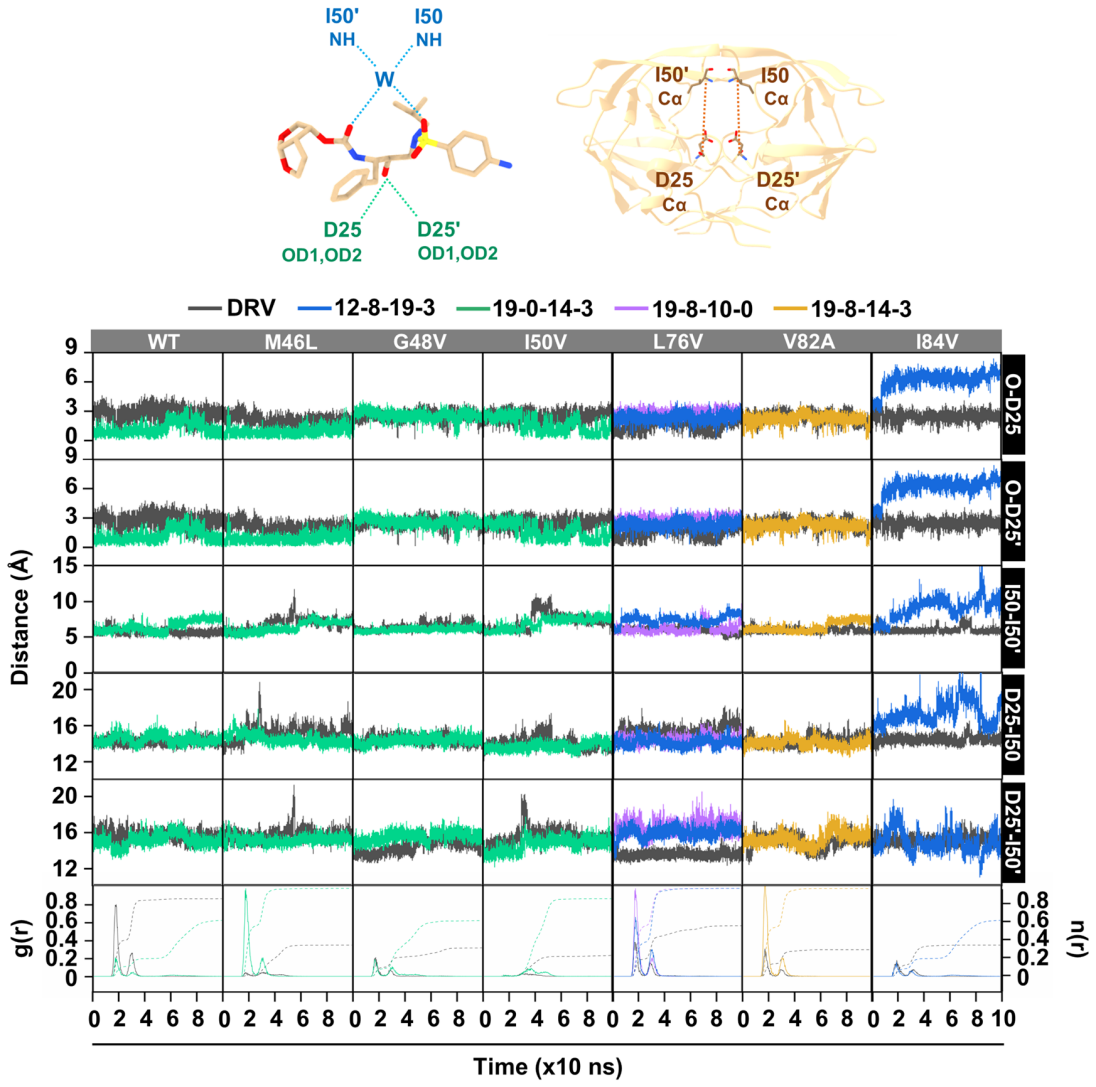
The three-dimensional structures of DRV and PR are represented in brown sticks, with dotted lines indicating distance. Water molecules are represented by W, while NH refers to the main chain amide group, Cα to the main chain carbon alpha, and OD1 and OD2 to the main chain oxygen group. The time series of O-D25, O-D25′, I50-I50′, D25-I50, and D25′-I50′ distances of the DRV/PRs and analogs/PRs systems are shown. The radial distribution function (RDF) of water oxygens around the NH nitrogen of DRV and analogs is calculated from the last 20-ns simulations of PRs solvated by TIP3P waters.

The ligand-binding stability of DRV and the five screened analogs in complex to WT HIV-1 PR and mutants were investigated from MD trajectory, as shown in Fig. 7. Previous studies have indicated that the OH of DRV forms an H-bond with catalytic residues D25 and D25′ of PR, maintaining a similar binding model^76,87,88^. Thus, the distance between the core structure of PRs (O) and D25 or D25′ (OD1, OD2) of DRV and analogs remained stable throughout the simulation, except in the **12–8–19–3**/I84V system. The O-D25 distance of PRs and DRV ranged from 2.0 to 2.8 Å, while the distance between PRs and the four analogs in six systems was from 1.9 to 2.9 Å, similar to the distance of O-D25′ between PRs and DRV or analogs systems. The results illustrated that the distance between PRs and D25 or D25′ of six PRs/analog systems (**19–0–14–3**/WT, **19–0–14–3**/M46L, **19–0–14–3**/G48V, **19–0–14–3**/I50V, **12–8–19–3**/L76V, **19–8–10–0**/L76V, and **19–8–14–3**/V82A) remained stable during the MD simulation compared to PRs/DRV systems. Therefore, the good interaction of analogs (Fig. 5 and 6) was estimated to be caused by the effectiveness of the designed substructures, not the core structure.

Comprehending the challenges associated with regulating the mobility of the HIV-PR flap can offer valuable insights into developing novel therapeutic inhibitors capable of disrupting flap opening and stabilizing them in the closed conformation. Thus, several prior computational studies have aimed to understand flap opening dynamics^89–92^. Previously, the interatomic distance between Ile50/50′, which is located at the flap tips of each monomer, was used to define the various conformations (closed, semi-open, and fully open conformations). Except for the system of **12–8–19–3**/I84V, the distance between the I50-I50′ residues of both the PRs/DRV and analog systems remained constant throughout the simulation, at approximately 6.2 Å. This finding is consistent with a previous study, which detected a closed state at approximately 5.8 Å in the presence of the inhibitor^93,94^. In the absence of the inhibitor, the HIV-PR structure undergoes a conformational change to a more flexible and semi-open state, allowing for proper flap reversal. Typically, PR mutations weaken the hydrophobic interactions related to I50 and I50′^15^; however, the results of the I50-I50′ distance reveal that potent analogs in the six systems play an essential role in maintaining the closed conformation not only of the WT but also of mutated PRs. Nonetheless, the I50-I50′ distance can be affected by both flap asymmetry and curling of the flap tip; therefore, it would be more reasonable to measure the distance between D25-I50 of chain A and D25′-I50′ of chain B in PRs^15,94–96^.

Note that, the D25-I50 and D25′-I50′ distances showed a similar pattern to the previous distance measurement. All six DRV/analog systems tested against PRs maintained a closed conformation (~ 14 Å) for both chains A and B. In contrast, the system of **12–8–19–3**/I84V exhibited an open conformation, characterized by higher fluctuations than the other systems (~ 18 Å). The ability of this system to stabilize a more open conformation would cause an increase in the volume of the active site cavity, which could have a vital impact on the binding of less favorable PIs^15,94^. Interestingly, water-mediated H-bonds were observed, forming attractive networks connecting a sulfonyl oxygen and hydroxyl group of DRV or analogs with the amino group on PRs^76,88^. The RDF results showed that around 0.6 to 0.9 water molecules were stabilized by the NH of analogs/PRs systems, whereas 0.3 to 0.8 water molecules of DRV/PRs systems were found. Therefore, all systems had water-mediated interactions with PR in the WT and mutated strains. Based on the distance analysis and RDF, the stable systems, except **12–8–19–3**/I84V, were further analyzed in depth for the interactions of overall compounds and each moiety.

### Binding pattern and interaction of DRV and the designed analogs

**Figure 8:**
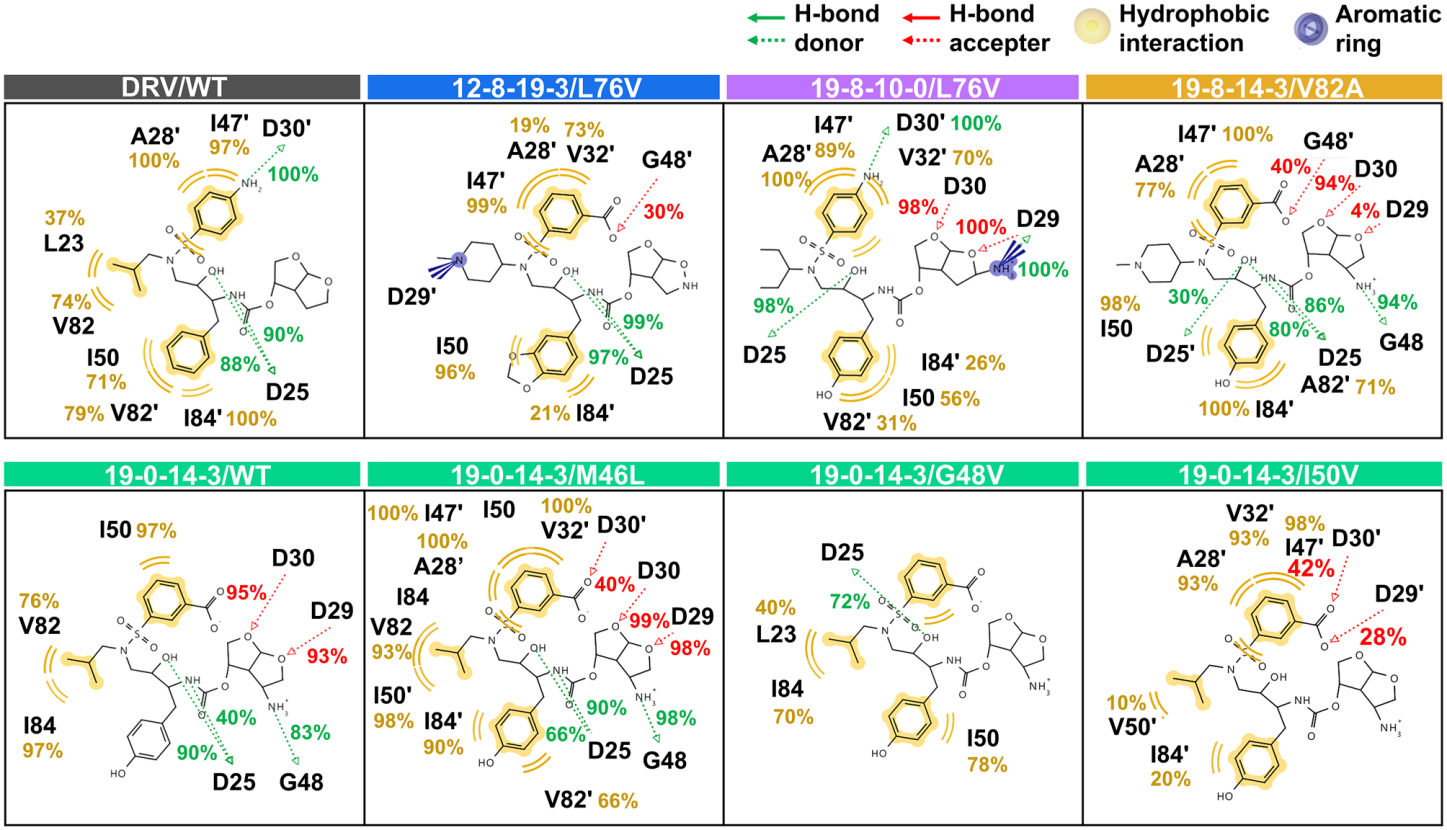
The vital interactions of DRV and potent analogs in complex with HIV-1 PR shown in the 2D pharmacophore models, which is analyzed from the last 20 ns-MD simulations. The green arrow, red arrow, yellow circle, and purple circle represent the pharmacophore features of hydrogen bond donor (HBD) and acceptor (HBA), hydrophobic interaction properties, and aromatic ring, respectively.

Several key interactions visualized by LigandScout 4.4.8^97^, consist of steric and electronic features shared by numerous active substances with similar biological targets. These features include positive and negatively charged groups, hydrophobic and aromatic areas, as well as HBD and HBA capabilities^98–100^. In this study, the MD trajectories of DRV and potent in complex with analogs HIV-1 PR were analyzed for pharmacophore, which is specialized in detecting ligand–protein interactions^101^. Figure 8 illustrates the 2D representative pharmacophore models (RPMS) of DRV and analogs (**12–8–19–3**, **19–0–14–3**, **19–8–10–0**, and **19–8–14–3**) extracted from the last 20 ns of the MD trajectories. Hydrogen bonding, HBD, or HBA, as well as hydrophobic interactions, were important chemical pharmacophore features for DRV and all analogs binding to HIV-1 PR through the core and substructures. The formation of H-bonds was investigated by defining criteria: the distance between the HBA and the HBD was ≤ 3.5 Å, and the angle of HBD − H···HBA was ≥ 120°. The percentage occupation of H-bonding of the DRV/analogs complexes is shown in Fig. 8. In the case of the reference drug, DRV showed hydrophobic interactions with L23, I50, V82, A28′, I47′, V82′, and I84′, similar to most analogs. However, there were some additional residues, V32′ for **12–8–19–3**/L76V and **19–8–10–0**/L76V systems, I84 for **19–0–14–3**/M46L and **19–0–14–3**/G48V systems, and V32′, I50′ for **19–0–14–3**/M46L system. By focusing on H-bond, **19–0–14–3**/M46L, **19–8–10–0**/L76V, and **19–8–14–3**/V82A showed a high occurrence of hydrogen bonding with the active binding residues D29 (99%), D29 (100%), and D30 (94%), respectively, and hydrophobic interaction with A28′ (77–100%), V32′ (70–100%), and I47′ (89–100%) in chain B. Moreover, an H-bond with G48 had been identified, while the **19–0–14–3**/M46L and **19–8–10–0**/L76V systems had a H-bond with D30′. Only the **19–8–14–3**/V82A complex was able to form a H-bond with G48′ (40%) operating as the active binding residue. Based on pharmacophore analysis, **19–0–14–3**/M46L, **19–8–10–0**/L76V, and **19–8–14–3**/V82A exhibit the highest number and percentage of pharmacophores. Therefore, these systems were selected for further exploration of the key binding residues compared to DRV in complex with M46L, L76V, and V82A.

### Key binding residue evaluation

**Figure 9:**
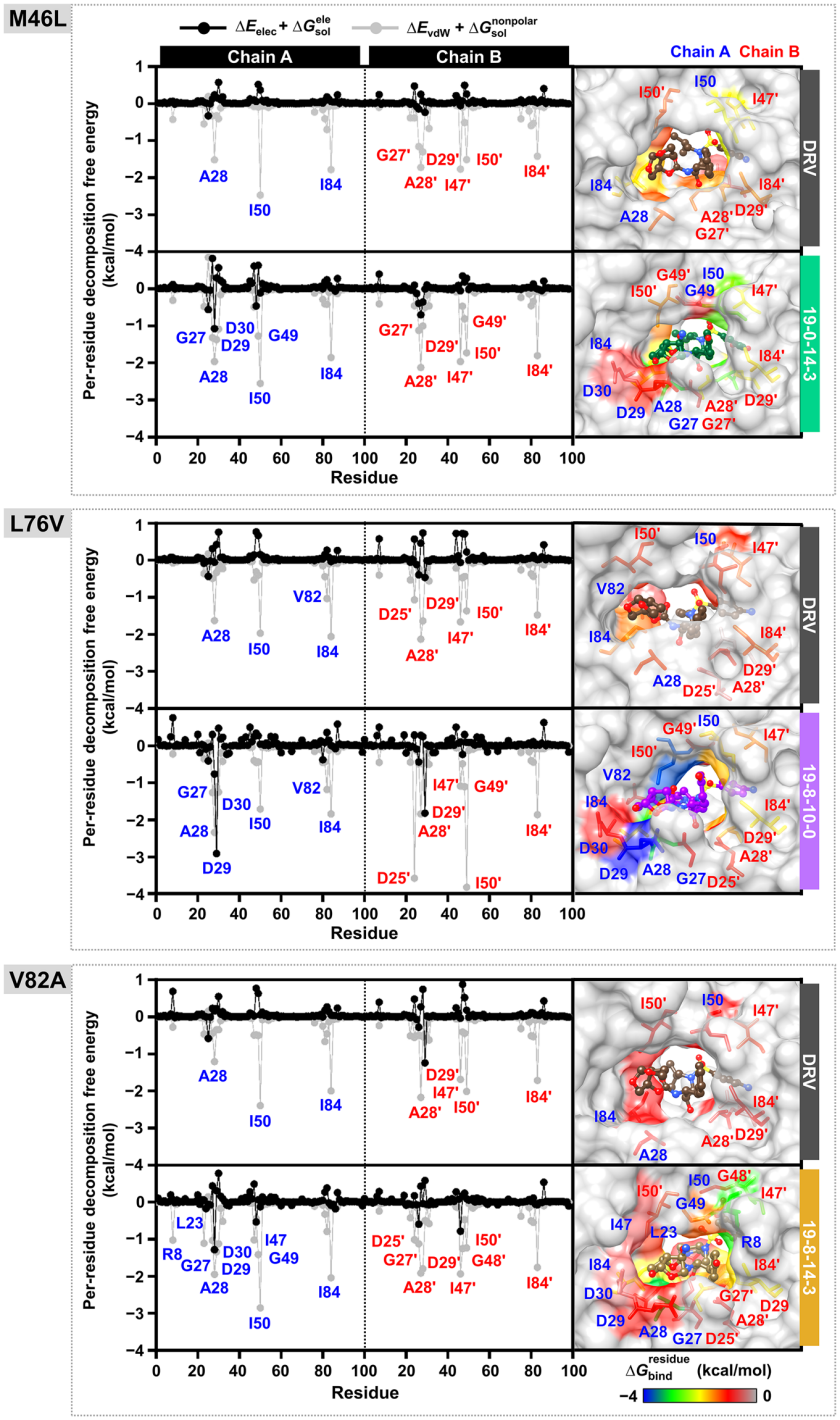
The values of electrostatic energy (Δ${E}_{elec}$ +Δ${G}_{sol}^{ele}$ , shown in black) and vdW (Δ${E}_{vdW}$ +Δ${G}_{sol}^{nonpolar}$, shown in grey) contributions from each residue of HIV-1 PR to the binding of darunavir (DRV) and its analogs were analyzed. The 3D structures that represent the ligand's binding orientation at the HIV-1 PR were selected from the clustered molecular dynamics (MD) snapshot. The residues that contribute to the ligand binding are colored based on their Δ*G*_*bind*_ residue values in chain A (black) and chain B (red). The residues are arranged in order of increasing energy, which is represented by colors ranging from gray to blue, respectively.

Figure 9 illustrates critical binding residue evaluation. The per-residue decomposition free energy (Δ ${G}_{bind}^{residue}$) calculation for **19–0–14–3**/M46L, **19–8–10–0**/L76V, and **19–8–14–3**/V82A compared to DRV in complex with M46L, L76V, and V82A by considering each modified position indicated that the aromatic rings at the P1 position of DRV and analogs were inserted into the S1 pocket of HIV-1 PR. These residues, such as I50 and I84 formed hydrophobic contacts and electrostatic interactions with DRV and three potent analogs, while G27 and G49 residues interacted with these analogs through H-bond. Only G27 was found to interact with DRV. These residues are consistent with previous studies that have identified significant binding residues such as D25, G27, A28, D29, and G49^76,102,103^.

For the P1′ position, R8 residue bound to the amino groups of **19–0–14–3** analog with attractive charge and electrostatic interaction in the docking process, while **19–8–14–3** analog represented vdW interaction with residues R8 and L23 in MD simulation. Both DRV and **19–8–10–0** interacted with I50′ in the L76V system via hydrophobic and electrostatic interactions. However, Δ ${G}_{bind}^{residue}$ values showed that **19–8–10–0** had a more effective interaction than DRV. The heterocyclic groups at the P2 position of DRV were inserted into the S2 pocket to form both vdW and electrostatic interactions with several residues. Interestingly, three potent analogs interacted with D29 and D30 through attractive charge, H-bond, and electrostatic interactions, resulting in more potent inhibitory effects against PRs than DRV. However, their contacts with residue A28 showed only vdW and electrostatic interactions.

The D25′ residue interacted with the aliphatic amine of **19–8–10–0** and **19–8–14–3** analogs through vdW, H-bond, and electrostatic interactions. The catalytic residues D25 and D25′ are crucial for acid–base catalysis in the aspartate active site, and most current HIV PIs mimic the substrate's transition state by using the amino group to interact with D25 and D25′ residues through H-bonds. However, only the heterocyclic amine group of **19–8–14–3** analog showed H-bonds^81,87,104–107^ with both residues G48 and G49′. The aromatic amines and carboxylic acid at the P2′ position of DRV and analogs bonded to the S2′ pocket, forming hydrophobic contacts and electrostatic interactions with residues A28′, D29′, and I47′. In the **19–8–14–3**/V82A system, there was also contact with the D29′ and G48′ residues via a H-bond, while the residue D30′ was found in the **19–8–10–0**/L76V system. The *bis*-THF moiety at P2′ contributed to high-binding affinity, forming strong H-bond with D29′ and D30′ residues^108,109^.

The vdW and electrostatic interactions of several residues, including A28, I50, I84, G49′, and I50′, were found to significantly contribute to the binding of inhibitors to both WT and mutated HIV-1 PR. Inhibitors that provide stronger interactions with other residues in the active site, such as G27, D25′, and G27′, via H-bonds^110–112^, are more potent. I50 and I50′ can form H-bonds with the inhibitor by utilizing water molecules in the active site, allowing the PR to assume an active conformation^104,113,114^. The residues D25 and D25′ also interact with the OH atoms of DRV and its analogs' core structure. Moreover, D30′ influences the NH and COOH of the P2′ moiety part, and R8 interacts with the amino group of the P1′ moiety part. Although electrostatic interactions primarily contribute to the complexation of HIV-1 PIs, H-bonds between DRV/analogs and their surrounding residues in the active site of HIV-1 PR can also play an important role in enzyme inhibition (Fig. S7 in Supporting Information).

## Conclusions

This study demonstrates the applicability of computer-assisted combinatorial chemistry methods for designing and in silico screening of DRV analogs as HIV-1 PR inhibitors. FMO-guided chemical substructure design proposes favorable chemical fragments to facilitate drug optimization, complemented by cascade screening methodologies using molecular docking techniques. Based on SIE and MM/GBSA calculations, MD simulations indicated that among the critical criteria are binding free energy, hotspot residue, and hydrogen bonding interaction. The susceptibility of **19–0–14–3**, **19–8–10–0**, and **19–8–14–3** analogs against PR was higher than DRV in WT and mutants HIV-1 PR, supported by the energy stabilization from the individual residues, suggesting that these designed analogs could be used to combat HIV as a global health issue. Moreover, the details on the crucial interactions for analog binding have also been elucidated, which is beneficial for future design. Additionally, the FMO/combinatorial programming-guided technique presented in this study could become an efficient tool for drug discovery.

### Supplementary Information

Supplementary Information.

## Data Availability

All of the designed DRV analogs, topology coordinates, and parameter datasets that support the conclusions of this article, as well as the cascaded structure-based drug design (SBDD) pipeline utilized for data analysis, are available in the Supporting Information files and at https://github.com/fahhathai/Tutorial_CombinatorialSubstructures.git.

## Code availability

An accompanying tutorial that involves a program for combining substructures is freely available at https://github.com/fahhathai/Tutorial_CombinatorialSubstructures/blob/main/Combinatorial_program.ipynb
