# Supplementary material for: FMO-guided design of darunavir analogs as HIV-1 protease inhibitors
Source: Sci Rep. 2024 Feb 13;14:3639. doi: 10.1038/s41598-024-53940-1 (PMC10864397; doi:10.1038/s41598-024-53940-1)
Supplement: Supplementary file 1 — Supplementary Information. [file 41598_2024_53940_MOESM1_ESM.docx]

**Supporting Information**

**FMO-Guided Design of Darunavir Analogs as HIV-1 Protease Inhibitors**

Hathaichanok Chuntakaruk^a,b^, Kowit Hengphasatporn^c*^, Yasuteru Shigeta^c^, Chanat Aonbangkhen^d^, Vannajan Sanghiran Lee^e^, Tanatorn Khotavivattana^d*^,Thanyada Rungrotmongkol^a,b*^ and Supot Hannongbua^f*^

^a^ Program in Bioinformatics and Computational Biology, Graduate School, Chulalongkorn University,

Bangkok 10330, Thailand

^b^Center of Excellence in Structural and Computational Biology, Department of Biochemistry, Faculty of

Science, Chulalongkorn University, Bangkok 10330, Thailand

^c^ Center for Computational Sciences, University of Tsukuba, 1-1-1 Tennodai, Tsukuba, Ibaraki 305-8577,

Japan

^d^ Center of Excellence in Natural Products Chemistry, Department of Chemistry, Faculty of Science,

Chulalongkorn University, Bangkok 10330, Thailand

^e^ Chemistry Department, Faculty of Science, University Malaya, Kuala Lumpur 50603, Malaysia

^f^ Center of Excellence in Computational Chemistry (CECC), Department of Chemistry, Faculty of Science,

Chulalongkorn University, Bangkok 10330, Thailand

E-mail: supot.h@chula.ac.th; thanyada.r@chula.ac.th; tanatorn.k@chula.ac.th

Phone: +66-2218-7707-6

**Figure S1.** 3D Structure of wild-type HIV-1 protease (HIV-1 PR) in complex with darunavir (PDB ID: 4LL3)

**Figure S2.** Flow chart of the DRV analogs designing protocol

**Figure S3.** Superimposition of the template of HIV-1 PR/DRV (pink) complex and the docked structure (blue) obtained from GOLD program visualized by UCSF Chimera package

**Figure S4.** Design 80 DRV analogs by modifying one functional group at P1, P1', P2, or P2' ssson the DRV scaffold. The scatter plot shows fitness scores compared to the fitness score of DRV (86.3).

**Figure S5.** Design 1,080 DRV analogs by modifying functional groups at P1, P1', P2, and P2' on DRV. The heatmap shows fitness scores compared to the fitness score of DRV (86.3).

**Table S1** PIE results from Fragment of DRV (F1, F1', F2, and F2')

| **F1** | ${\Delta E}_{IJ}^{ES}$ | | ${\Delta E}_{IJ}^{CT+mix}$ | | | ${\Delta E}_{IJ}^{DI}$ | | | ${\Delta E}_{IJ}^{EX}$ | | ${\Delta G}_{Sol}^{PCM}$ | | | PIE | | |
| --- | --- | --- | --- | --- | --- | --- | --- | --- | --- | --- | --- | --- | --- | --- | --- | --- |
| **L10** | | 0 | 0 | | 0 | | | 0 | | | 0 | | 0 | | |  |
| **L23** | | 0 | 0 | | 0 | | | 0 | | | 0 | | 0 | | |  |
| **L24** | | 0 | 0 | | 0 | | | 0 | | | 0 | | 0 | | |  |
| **D25** | | 0 | 0 | | 0 | | | 0 | | | 0 | | 0 | | |  |
| **T26** | | -0.153 | 0 | | -0.116 | | | 0 | | | 0.037 | | -0.116 | | |  |
| **G27** | | 0.417 | -0.05 | | 0.175 | | | 0.108 | | | -0.029 | | 0.175 | | |  |
| **A28** | | -1.055 | -0.952 | | -2.731 | | | 0.7 | | | 0.096 | | -2.731 | | |  |
| **D29** | | 0 | 0 | | 0 | | | 0 | | | 0 | | 0 | | |  |
| **D30** | | 0 | 0 | | 0 | | | 0 | | | 0 | | 0 | | |  |
| **V32** | | 0 | 0 | | 0 | | | 0 | | | 0 | | 0 | | |  |
| **M46** | | 0 | 0 | | 0 | | | 0 | | | 0 | | 0 | | |  |
| **I47** | | 0 | 0 | | 0 | | | 0 | | | 0 | | 0 | | |  |
| **G48** | | -0.279 | 0 | | -0.241 | | | 0 | | | 0.039 | | -0.241 | | |  |
| **G49** | | 1.063 | -0.422 | | 0.425 | | | 0.757 | | | -0.13 | | 0.425 | | |  |
| **I50** | | -1.29 | -0.524 | | -2.392 | | | 1.851 | | | 0.199 | | -2.392 | | |  |
| **T80** | | 0 | 0 | | 0 | | | 0 | | | 0 | | 0 | | |  |
| **P81** | | 0 | 0 | | 0 | | | 0 | | | 0 | | 0 | | |  |
| **V82** | | 0 | 0 | | 0 | | | 0 | | | 0 | | 0 | | |  |
| **N83** | | 0 | 0 | | 0 | | | 0 | | | 0 | | 0 | | |  |
| **I84** | | 0 | 0 | | 0 | | | 0 | | | 0 | | 0 | | |  |
| **Q7'** | | 0 | 0 | | 0 | | | 0 | | | 0 | | 0 | | |  |
| **R8'** | | 0 | 0 | | 0 | | | 0 | | | 0 | | 0 | | |  |
| **L10'** | | 0.616 | 0 | | 0.348 | | | 0 | | | -0.268 | | 0.348 | | |  |
| **L23'** | | -1.375 | -0.163 | | -1.524 | | | 0.124 | | | 0.525 | | -1.524 | | |  |
| **L24'** | | 0 | 0 | | 0 | | | 0 | | | 0 | | 0 | | |  |
| **D25'** | | 0.89 | -0.274 | | -0.091 | | | 0.166 | | | -0.177 | | -0.091 | | |  |
| **G27'** | | 0 | 0 | | 0 | | | 0 | | | 0 | | 0 | | |  |
| **A28'** | | 0 | 0 | | 0 | | | 0 | | | 0 | | 0 | | |  |
| **D29'** | | 0 | 0 | | 0 | | | 0 | | | 0 | | 0 | | |  |
| **D30'** | | 0 | 0 | | 0 | | | 0 | | | 0 | | 0 | | |  |
| **V32'** | | 0 | 0 | | 0 | | | 0 | | | 0 | | 0 | | |  |
| **K45'** | | 0 | 0 | | 0 | | | 0 | | | 0 | | 0 | | |  |
| **I47'** | | 0 | 0 | | 0 | | | 0 | | | 0 | | 0 | | |  |
| **G48'** | | 0 | 0 | | 0 | | | 0 | | | 0 | | 0 | | |  |
| **G49'** | | 0 | 0 | | 0 | | | 0 | | | 0 | | 0 | | |  |
| **I50'** | | 0 | 0 | | 0 | | | 0 | | | 0 | | 0 | | |  |
| **T80'** | | -0.039 | 0 | | -0.124 | | | 0 | | | -0.085 | | -0.124 | | |  |
| **P81'** | | -0.573 | -0.526 | | -1.61 | | | 1.071 | | | -0.083 | | -1.61 | | |  |
| **V82'** | | -0.457 | -0.6 | | -1.752 | | | 1.246 | | | 0.098 | | -1.752 | | |  |
| **N83'** | | 0 | 0 | | 0 | | | 0 | | | 0 | | 0 | | |  |
| **I84'** | | 0.188 | -0.21 | | -0.782 | | | 0.172 | | | -0.099 | | -0.782 | | |  |
| **R87'** | | 0.347 | 0 | | 0.291 | | | 0 | | | -0.056 | | 0.291 | | |  |
| **WAT** | | 1.229 | -0.245 | | 0.81 | | | 0.046 | | | 0.077 | | 0.81 | | |  |
| **F1'** | |  |  | |  | | |  | | |  | |  | | |  |
| **L10** | | 0 | 0 | 0 | | | 0 | | | 0 | | 0 | | |  |  |
| **L23** | | -1.669 | -0.319 | -1.085 | | | 1.633 | | | 0.299 | | -1.142 | | |  |  |
| **L24** | | 0 | 0 | 0 | | | 0 | | | 0 | | 0 | | |  |  |
| **D25** | | -1.508 | -0.687 | -0.786 | | | 0.215 | | | -0.019 | | -2.786 | | |  |  |
| **T26** | | 0 | 0 | 0 | | | 0 | | | 0 | | 0 | | |  |  |
| **G27** | | 0 | 0 | 0 | | | 0 | | | 0 | | 0 | | |  |  |
| **A28** | | 0 | 0 | 0 | | | 0 | | | 0 | | 0 | | |  |  |
| **D29** | | 0 | 0 | 0 | | | 0 | | | 0 | | 0 | | |  |  |
| **D30** | | 0 | 0 | 0 | | | 0 | | | 0 | | 0 | | |  |  |
| **V32** | | 0 | 0 | 0 | | | 0 | | | 0 | | 0 | | |  |  |
| **M46** | | 0 | 0 | 0 | | | 0 | | | 0 | | 0 | | |  |  |
| **I47** | | 0 | 0 | 0 | | | 0 | | | 0 | | 0 | | |  |  |
| **G48** | | 0 | 0 | 0 | | | 0 | | | 0 | | 0 | | |  |  |
| **G49** | | 0 | 0 | 0 | | | 0 | | | 0 | | 0 | | |  |  |
| **I50** | | 0 | 0 | 0 | | | 0 | | | 0 | | 0 | | |  |  |
| **T80** | | 0.031 | -0.031 | -0.054 | | | -0.001 | | | 0.009 | | -0.047 | | |  |  |
| **P81** | | -0.176 | -0.035 | -0.138 | | | 0.004 | | | -0.005 | | -0.351 | | |  |  |
| **V82** | | -0.38 | -0.072 | -0.327 | | | 0.05 | | | 0.036 | | -0.693 | | |  |  |
| **N83** | | 0 | 0 | 0 | | | 0 | | | 0 | | 0 | | |  |  |
| **I84** | | -0.077 | -0.337 | -1.658 | | | 1.129 | | | -0.051 | | -0.995 | | |  |  |
| **Q7'** | | 0 | 0 | 0 | | | 0 | | | 0 | | 0 | | |  |  |
| **R8'** | | 0 | 0 | 0 | | | 0 | | | 0 | | 0 | | |  |  |
| **L10'** | | 0 | 0 | 0 | | | 0 | | | 0 | | 0 | | |  |  |
| **L23'** | | 0 | 0 | 0 | | | 0 | | | 0 | | 0 | | |  |  |
| **L24'** | | -0.13 | 0 | 0 | | | 0 | | | -0.018 | | -0.148 | | |  |  |
| **D25'** | | -0.006 | -0.024 | -0.075 | | | -0.001 | | | 0.018 | | -0.087 | | |  |  |
| **G27'** | | 0.547 | -0.01 | -0.123 | | | 0.016 | | | -0.038 | | 0.392 | | |  |  |
| **A28'** | | 0 | 0 | 0 | | | 0 | | | 0 | | 0 | | |  |  |
| **D29'** | | 0 | 0 | 0 | | | 0 | | | 0 | | 0 | | |  |  |
| **D30'** | | 0 | 0 | 0 | | | 0 | | | 0 | | 0 | | |  |  |
| **V32'** | | 0 | 0 | 0 | | | 0 | | | 0 | | 0 | | |  |  |
| **K45'** | | 0 | 0 | 0 | | | 0 | | | 0 | | 0 | | |  |  |
| **I47'** | | 0 | 0 | 0 | | | 0 | | | 0 | | 0 | | |  |  |
| **G48'** | | 0.118 | 0 | 0 | | | 0 | | | -0.109 | | 0.008 | | |  |  |
| **G49'** | | -0.877 | -0.11 | -0.094 | | | 0.002 | | | 0.429 | | -0.651 | | |  |  |
| **I50'** | | 0.402 | -0.128 | -0.418 | | | 0.039 | | | 0.002 | | -0.102 | | |  |  |
| **T80'** | | 0 | 0 | 0 | | | 0 | | | 0 | | 0 | | |  |  |
| **P81'** | | 0 | 0 | 0 | | | 0 | | | 0 | | 0 | | |  |  |
| **V82'** | | 0 | 0 | 0 | | | 0 | | | 0 | | 0 | | |  |  |
| **N83'** | | 0 | 0 | 0 | | | 0 | | | 0 | | 0 | | |  |  |
| **I84'** | | 0 | 0 | 0 | | | 0 | | | 0 | | 0 | | |  |  |
| **R87'** | | 0.29 | 0 | 0 | | | 0 | | | 0.196 | | 0.487 | | |  |  |
| **WAT** | | 0.179 | -0.346 | -0.277 | | | 0.127 | | | 0.016 | | -0.3 | | |  |  |
| **F2** | |  |  |  | | |  | | |  | |  | | |  |  |
| **L10** | | 0 | 0 | 0 | | | 0 | | | 0 | | 0 | | |  |  |
| **L23** | | 0 | 0 | 0 | | | 0 | | | 0 | | 0 | | |  |  |
| **L24** | | 0.465 | 0 | 0 | | | 0 | | | 0.089 | | 0.554 | | |  |  |
| **D25** | | -7.149 | -1.293 | -1.774 | | | 1.492 | | | -0.687 | | -9.41 | | |  |  |
| **T26** | | 0 | 0 | 0 | | | 0 | | | 0 | | 0 | | |  |  |
| **G27** | | -1.339 | -1.072 | -0.989 | | | 1.709 | | | 0.11 | | -1.58 | | |  |  |
| **A28** | | -5.068 | -2.48 | -5.646 | | | 3.812 | | | 0.003 | | -9.378 | | |  |  |
| **D29** | | -2.866 | -2.547 | -6.258 | | | 8.399 | | | -2.239 | | -5.512 | | |  |  |
| **D30** | | -3.577 | -2.163 | -4.076 | | | 3.794 | | | -1.976 | | -7.998 | | |  |  |
| **V32** | | 0.848 | -0.156 | -0.97 | | | 0.515 | | | -0.255 | | -0.018 | | |  |  |
| **M46** | | 0.049 | 0 | 0 | | | 0 | | | -0.047 | | 0.002 | | |  |  |
| **I47** | | 0.097 | -0.35 | -1.239 | | | 0.27 | | | 0.14 | | -1.082 | | |  |  |
| **G48** | | 0.758 | -0.048 | -0.19 | | | -0.002 | | | -0.346 | | 0.172 | | |  |  |
| **G49** | | -1.027 | -0.909 | -1.708 | | | 0.551 | | | 0.206 | | -2.886 | | |  |  |
| **I50** | | -3.156 | -0.07 | -0.34 | | | 0 | | | 0.208 | | -3.358 | | |  |  |
| **T80** | | 0 | 0 | 0 | | | 0 | | | 0 | | 0 | | |  |  |
| **P81** | | 0 | 0 | 0 | | | 0 | | | 0 | | 0 | | |  |  |
| **V82** | | 0 | 0 | 0 | | | 0 | | | 0 | | 0 | | |  |  |
| **N83** | | 0.452 | 0 | 0 | | | 0 | | | 0.051 | | 0.503 | | |  |  |
| **I84** | | -0.395 | -0.149 | -1.359 | | | 0.771 | | | -0.156 | | -1.287 | | |  |  |
| **Q7'** | | -1.598 | 0 | 0 | | | 0 | | | 0.584 | | -1.015 | | |  |  |
| **R8'** | | -3.409 | -0.059 | -0.232 | | | 0 | | | 0.102 | | -3.598 | | |  |  |
| **L10'** | | 0 | 0 | 0 | | | 0 | | | 0 | | 0 | | |  |  |
| **L23'** | | 0 | 0 | 0 | | | 0 | | | 0 | | 0 | | |  |  |
| **L24'** | | -0.998 | 0 | 0 | | | 0 | | | -0.15 | | -1.148 | | |  |  |
| **D25'** | | -24.299 | -7.677 | -3.285 | | | 21.649 | | | -0.039 | | -13.65 | | |  |  |
| **G27'** | | 0 | 0 | 0 | | | 0 | | | 0 | | 0 | | |  |  |
| **A28'** | | -0.08 | 0.008 | -0.064 | | | -0.002 | | | 0.12 | | -0.018 | | |  |  |
| **D29'** | | 0 | 0 | 0 | | | 0 | | | 0 | | 0 | | |  |  |
| **D30'** | | 0 | 0 | 0 | | | 0 | | | 0 | | 0 | | |  |  |
| **V32'** | | 0 | 0 | 0 | | | 0 | | | 0 | | 0 | | |  |  |
| **K45'** | | 0 | 0 | 0 | | | 0 | | | 0 | | 0 | | |  |  |
| **I47'** | | 0 | 0 | 0 | | | 0 | | | 0 | | 0 | | |  |  |
| **G48'** | | 0 | 0 | 0 | | | 0 | | | 0 | | 0 | | |  |  |
| **G49'** | | -1.114 | 0 | 0 | | | 0 | | | 0.095 | | -1.019 | | |  |  |
| **I50'** | | -1.888 | -0.794 | -1.936 | | | 1.076 | | | 0.002 | | -3.54 | | |  |  |
| **T80'** | | 0 | 0 | 0 | | | 0 | | | 0 | | 0 | | |  |  |
| **P81'** | | 0 | 0 | 0 | | | 0 | | | 0 | | 0 | | |  |  |
| **V82'** | | 0 | 0 | 0 | | | 0 | | | 0 | | 0 | | |  |  |
| **N83'** | | 0 | 0 | 0 | | | 0 | | | 0 | | 0 | | |  |  |
| **I84'** | | 0 | 0 | 0 | | | 0 | | | 0 | | 0 | | |  |  |
| **R87'** | | 0.577 | 0 | 0 | | | 0 | | | 0.776 | | 1.353 | | |  |  |
| **WAT** | | -15.771 | -3.79 | -2.367 | | | 9.776 | | | 0.075 | | -12.077 | | |  |  |
| **F2'** | |  |  |  | | |  | | |  | |  | | |  |  |
| **L10** | | -1.504 | 0 | 0 | | | 0 | | | 1.189 | | -0.316 | | |  |  |
| **L23** | | 2.358 | -0.004 | -0.183 | | | 0 | | | -1.514 | | 0.657 | | |  |  |
| **L24** | | 0 | 0 | 0 | | | 0 | | | 0 | | 0 | | |  |  |
| **D25** | | -7.592 | -0.904 | -2.031 | | | 1.876 | | | 1.633 | | -7.018 | | |  |  |
| **T26** | | 0 | 0 | 0 | | | 0 | | | 0 | | 0 | | |  |  |
| **G27** | | 0.943 | 0.073 | -0.15 | | | -0.001 | | | -0.122 | | 0.743 | | |  |  |
| **A28** | | 0 | 0 | 0 | | | 0 | | | 0 | | 0 | | |  |  |
| **D29** | | 0 | 0 | 0 | | | 0 | | | 0 | | 0 | | |  |  |
| **D30** | | 0 | 0 | 0 | | | 0 | | | 0 | | 0 | | |  |  |
| **V32** | | 0 | 0 | 0 | | | 0 | | | 0 | | 0 | | |  |  |
| **M46** | | 0 | 0 | 0 | | | 0 | | | 0 | | 0 | | |  |  |
| **I47** | | 0 | 0 | 0 | | | 0 | | | 0 | | 0 | | |  |  |
| **G48** | | 0 | 0 | 0 | | | 0 | | | 0 | | 0 | | |  |  |
| **G49** | | -0.96 | 0.021 | -0.009 | | | -0.001 | | | -0.409 | | -1.359 | | |  |  |
| **I50** | | -3.976 | -1.625 | -4.954 | | | 3.739 | | | -0.677 | | -7.494 | | |  |  |
| **T80** | | 0 | 0 | 0 | | | 0 | | | 0 | | 0 | | |  |  |
| **P81** | | 0 | 0 | 0 | | | 0 | | | 0 | | 0 | | |  |  |
| **V82** | | 0 | 0 | 0 | | | 0 | | | 0 | | 0 | | |  |  |
| **N83** | | 0 | 0 | 0 | | | 0 | | | 0 | | 0 | | |  |  |
| **I84** | | 0 | 0 | 0 | | | 0 | | | 0 | | 0 | | |  |  |
| **Q7'** | | 0 | 0 | 0 | | | 0 | | | 0 | | 0 | | |  |  |
| **R8'** | | 0 | 0 | 0 | | | 0 | | | 0 | | 0 | | |  |  |
| **L10'** | | 0 | 0 | 0 | | | 0 | | | 0 | | 0 | | |  |  |
| **L23'** | | 0 | 0 | 0 | | | 0 | | | 0 | | 0 | | |  |  |
| **L24'** | | 0.191 | 0 | 0 | | | 0 | | | 0.051 | | 0.242 | | |  |  |
| **D25'** | | 1.403 | -0.533 | -2.473 | | | 3.27 | | | -0.086 | | 1.581 | | |  |  |
| **G27'** | | 0.178 | -0.078 | -0.16 | | | 0.003 | | | -0.004 | | -0.061 | | |  |  |
| **A28'** | | 0.34 | -1.306 | -3.339 | | | 2.409 | | | 0.418 | | -1.478 | | |  |  |
| **D29'** | | -3.95 | 0.014 | -1.458 | | | 0.155 | | | 2.252 | | -2.987 | | |  |  |
| **D30'** | | -16.086 | -2.642 | -3.992 | | | 5.185 | | | 6.275 | | -11.26 | | |  |  |
| **V32'** | | 0.549 | -0.19 | -0.468 | | | 0.022 | | | 1.42 | | 1.332 | | |  |  |
| **K45'** | | 8.402 | -0.051 | -0.087 | | | -0.001 | | | -6.699 | | 1.563 | | |  |  |
| **I47'** | | -1.857 | -0.783 | -2.565 | | | 1.046 | | | 0.274 | | -3.885 | | |  |  |
| **G48'** | | 2.316 | -0.01 | -0.194 | | | 0 | | | -1.023 | | 1.089 | | |  |  |
| **G49'** | | -3.855 | -1.511 | -2.641 | | | 2.055 | | | 1.633 | | -4.319 | | |  |  |
| **I50'** | | -5.991 | 0.512 | -0.634 | | | 0.02 | | | -0.238 | | -6.331 | | |  |  |
| **T80'** | | 0 | 0 | 0 | | | 0 | | | 0 | | 0 | | |  |  |
| **P81'** | | 0 | 0 | 0 | | | 0 | | | 0 | | 0 | | |  |  |
| **V82'** | | 0 | 0 | 0 | | | 0 | | | 0 | | 0 | | |  |  |
| **N83'** | | 1.054 | 0 | 0 | | | 0 | | | 0.079 | | 1.133 | | |  |  |
| **I84'** | | -0.502 | -0.351 | -1.849 | | | 1.251 | | | -0.242 | | -1.692 | | |  |  |
| **R87'** | | 0.342 | 0 | 0 | | | 0 | | | -0.03 | | 0.312 | | |  |  |
| **WAT** | | -13.904 | -3.293 | -2.511 | | | 12.442 | | | -0.241 | | -7.507 | | |  |  |

**Table S2.** The relative fitness scores of DRV and 55 analogs in wild type and mutants

**Figure S6.** All-atom RMSD, # H-bonds, and # atom contacts of DRV in complex with WT HIV-1 PR plotted along the 100-ns MD simulations in triplicates.

**Table S3**. MM/GBSA binding free energy and energetic components (kcal/mol) of DRV toward WT HIV-1 PR in triplicates compared to SIE binding free energy.

|  | **DRV (n1)** | **DRV (n2)** | **DRV (n3)** |
| --- | --- | --- | --- |
| **∆*E_ele_*** | −47.3 ± 0.4 | −47.0 ± 0.6 | −42.1 ± 0.7 |
| **∆*E_vdW_*** | −58.1 ± 0.2 | −57.0 ± 0.3 | −56.5 ± 0.3 |
| **∆*E_MM_*** | −105.4 ± 0.6 | −104.0 ± 0.9 | −98.6 ± 1.0 |
| **∆*G_nonpolar,sol_*** | −8.0 ± 0.2 | −8.2 ± 0.1 | −8.0 ± 0.1 |
| **∆*G_ele,sol_*** | 65.1 ± 0.4 | 64.9 ± 0.4 | 61.3 ± 0.6 |
| **∆*G_sol_*** | 58.9 ± 0.4 | 60.6 ± 0.4 | 53.3 ± 0.1 |
| **∆*G_ele,sol_ + ∆E_ele_*** | 17.8 ± 0.8 | 17.9 ± 1.0 | 19.2 ± 1.3 |
| **∆*G_nonpolar,sol_ + ∆E_vdW_*** | −66.1 ± 0.4 | −65.2 ± 0.4 | −64.5 ± 0.4 |
| **−*T∆S*** | −30.4 ± 0.5 | −27.9 ± 1.0 | −29.4 ± 0.2 |
| ${\text{∆}\text{G}}_{\text{bind}}^{\text{MM/GBSA}}$ | **−16.1 ± 1.0** | **−15.5 ± 1.2** | **−15.9 ± 1.3** |
| ${\text{∆}\text{G}}_{\text{bind}}^{\text{SIE}}$ | **−9.9 ± 0.3** | **−9.5 ± 0.1** | **−9.7 ± 0.4** |

**Figure S7.** The 3D and representative pharmacophore models (RPMs) of the first representative frame of DRV/potent analogs at last 20 ns.
